# Safety and Efficacy of Metformin for Idiopathic Intracranial Hypertension. A U.S-Based Real-World Data Retrospective Multicenter Cohort Study

**DOI:** 10.71079/2024001001

**Published:** 2024-12-28

**Authors:** Ahmed Y. Azzam, Mahmoud Nassar, Ahmed Saad Al Zomia, Adam Elswedy, Mahmoud M. Morsy, Adham A. Mohamed, Osman Elamin, Omar S. Elsayed, Mohammed A. Azab, Muhammed Amir Essibayi, Jin Wu, Adam A. Dmytirw, David J. Altschul

**Affiliations:** 1- Montefiore-Einstein Cerebrovascular Research Lab, Montefiore Medical Center, Albert Einstein College of Medicine, Bronx, NY, USA.; 2- Department of Medicine, Jacobs School of Medicine and Biomedical Sciences, University at Buffalo, New York, USA.; 3- College of Medicine, King Khalid University, Abha, Saudi Arabia.; 4- Faculty of Medicine, October 6 University, Giza, Egypt.; 5- Cairo University Hospitals, Cairo University, Cairo, Egypt.; 6- Department of Neurosurgery, Jordan Hospital, Amman, Jordan.; 7- Department of Neurosurgery, Cleveland Clinic Foundation, Cleveland, OH, USA.; 8- Department of Neurological Surgery, Montefiore Medical Center, Albert Einstein College of Medicine, Bronx, NY, USA.; 9- National Institute of Neurological Disorders and Stroke, National Institutes of Health, Bethesda, Maryland, USA.; 10- Neuroendovascular Program, Massachusetts General Hospital, Harvard Medical School, Boston, MA, USA.; 11- Neurovascular Centre, Divisions of Therapeutic Neuroradiology & Neurosurgery, St. Michael’s Hospital, University of Toronto, Toronto, ON, Canada.

**Keywords:** Idiopathic Intracranial Hypertension, Pseudotumor Cerebri, Metformin, Metabolic Syndrome

## Abstract

**Introduction:**

Managing idiopathic intracranial hypertension (IIH) is challenging due to limited treatment options. This study evaluates metformin as a potential therapy for IIH, examining its impact on disease outcomes and safety.

**Methods:**

We performed a retrospective cohort study using the TriNetX database, covering data from 2009 to August 2024. The study included IIH patients, excluding those with other causes of raised intracranial pressure or pre-existing diabetes. Propensity score matching adjusted for age, sex, race, ethnicity, Hemoglobin A1C, and baseline BMI at metformin initiation. We assessed outcomes up to 24 months.

**Results:**

Initially, 1,268 patients received metformin and 49,262 served as controls, showing disparities in various parameters. After matching, both groups consisted of 1,267 patients each. Metformin users had significantly lower risks of papilledema, headache, and refractory IIH at all follow-ups (p<0.0001). They also had fewer spinal punctures and reduced acetazolamide use. BMI reductions were more significant in the metformin group from 6 months onward (p<0.0001), with benefits persisting regardless of BMI changes. Metformin’s safety profile was comparable to the control group.

**Conclusions:**

The study indicates metformin’s potential as a disease-modifying treatment in IIH, with improvements across multiple outcomes independent of weight loss. This suggests complex mechanisms at play, supporting further research through prospective clinical trials to confirm metformin’s role in IIH management and its mechanisms of action.

## 1. Introduction

The current standard of care for idiopathic intracranial hypertension (IIH) focuses on reducing intracranial pressure (ICP) and preserving visual function [1, 2]. Weight loss remains the cornerstone of therapy, with studies demonstrating significant improvements in ICP and clinical outcomes following a 5–10% reduction in body weight [3, 4]. The Idiopathic Intracranial Hypertension Weight Trial (IIH: WT) provided Class I evidence that bariatric surgery is superior to community weight management programs in reducing ICP and improving quality of life [5]. Pharmacological management primarily involves acetazolamide, a carbonic anhydrase inhibitor that decreases cerebrospinal fluid (CSF) production. The landmark Idiopathic Intracranial Hypertension Treatment Trial (IIHTT) established acetazolamide’s efficacy in improving visual field function and reducing ICP when combined with a low-sodium weight reduction diet [6]. Other therapeutic approaches include topiramate, which offers the dual benefit of ICP reduction and migraine prophylaxis, and surgical interventions such as CSF diversion procedures or optic nerve sheath fenestration for medically refractory cases [7].

Despite these interventions, the management of IIH remains challenging, with a considerable proportion of patients experiencing refractory or recurrent disease [8]. Refractory IIH is defined as persistent or worsening symptoms and signs (including headaches, papilledema, and visual outcomes) despite maximal medical therapy (usually consisting of weight loss interventions and maximum tolerated doses of acetazolamide) for at least three months. Recurrent disease refers to the return of IIH symptoms and signs after a period of remission, often requiring reinitiation or intensification of therapy [8]. Many patients struggle to achieve or maintain weight loss, particularly through non-surgical means. The side effect profile of acetazolamide, including paresthesia, dysgeusia, and fatigue, often limits its long-term use or dose escalation [9]. Furthermore, a significant proportion of patients experience a plateau in their clinical improvement or require multiple interventions to maintain remission [10]. The lack of targeted therapies addressing the underlying pathophysiology of IIH, particularly the complex interplay between adipose dysfunction, CSF dynamics, and metabolic dysregulation, has hindered progress in disease modification and long-term outcomes [11].

**Figure 1:**
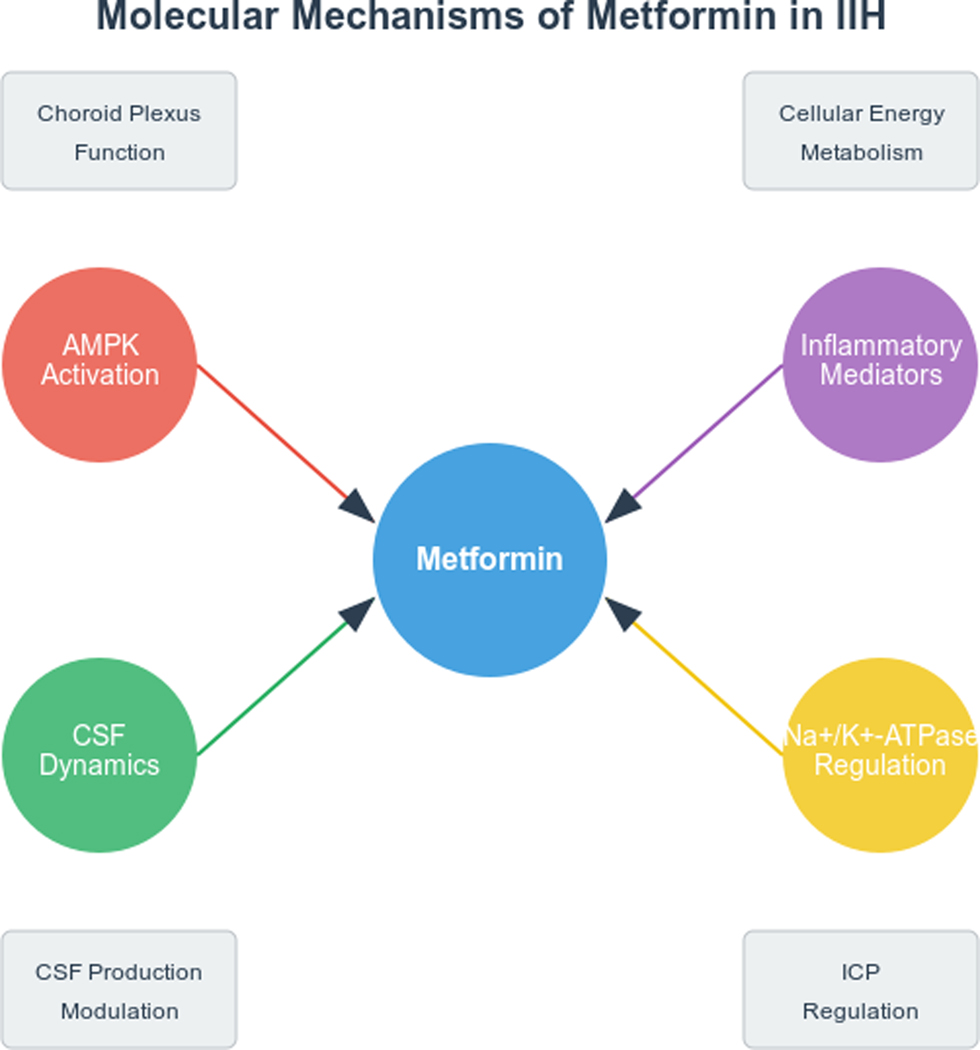
Mechanisms of Action of Metformin in IIH.

The latest literature evidence has highlighted the unmet need for novel treatment approaches for IIH. Metformin, a biguanide antidiabetic agent, has demonstrated pleiotropic effects beyond glucose control, including modulation of adipose tissue function and reduction of CSF secretion [12]. Preclinical studies have shown that metformin can lower ICP through AMP-activated protein kinase (AMPK)-dependent inhibition of Na+/K+-ATPase at the choroid plexus, suggesting a direct mechanism for CSF production reduction [13]. This effect is particularly intriguing given the recent evidence implicating choroid plexus hypersecretion in IIH pathogenesis [14]. Additionally, metformin’s effects on weight loss, insulin sensitivity, and adipokine profiles may address key pathogenic factors in CSF disorders such as hydrocephalus in rodent models, offering a potential approach to related diseases management in certain phenotypes [15] (Figure 1).

The potential of metformin in IIH is further supported by its established safety profile and its ability to mitigate components of metabolic syndrome [16], which are increasingly recognized as contributors to IIH pathophysiology [17]. To address this knowledge gap and explore metformin’s potential as a disease-modifying therapy for IIH, we are conducting a multicenter, retrospective cohort study utilizing the TriNetX database. This large-scale, real-world evidence approach allows for the assessment of metformin’s impact on IIH outcomes across diverse clinical settings in the United States, providing valuable insights into its safety and efficacy in a large patient cohort. Our study aims to evaluate the effects of metformin on IIH-related symptoms, healthcare utilization, and long-term disease progression, offering a robust foundation for future prospective clinical trials. By leveraging this comprehensive dataset, we seek to elucidate metformin’s potential role in expanding the therapeutic armamentarium for IIH, potentially offering a novel, mechanistically targeted approach to this challenging condition.

## 2. Methods

Our study utilized data from the expansive TriNetX Research Network, through the global collaborative network database [18], which contains around 197 million electronic health records aggregated from more than 160 healthcare organizations, primarily located in the United States. This comprehensive dataset includes a wide range of patient-level information, such as demographic characteristics, diagnoses, treatments, procedures, and outcomes, all coded using standard medical classification systems like the International Classification of Diseases, 10th Revision (ICD-10) and Current Procedural Terminology (CPT). Researchers can access this extensive real-world data through the secure TriNetX platform to conduct observational studies. Notably, the dataset is regularly updated, ensuring access to the most current and comprehensive healthcare information available. The study protocol was approved by the Institutional Review Board at the Jacobs School of Medicine and Biomedical Sciences, University at Buffalo, NY, USA (STUDY00008628).

We performed a retrospective analysis of the TriNetX data from 2009 to August 2024 (the timeframe associated with individuals with our inclusion and exclusion criteria in the TriNetX database), focusing on patients diagnosed with IIH. Patients were included if they had a primary diagnosis of IIH (ICD-10 code: G93.2), were aged 18 years or older, had at least one recorded BMI measurement and had a minimum follow-up period of 1 month. We excluded patients with pre-existing type 1 or type 2 diabetes mellitus (ICD-10 codes: E10.*, E11.*), prediabetes (ICD-10: R73.03), or HbA1c ≥ 6.5%. Additional exclusion criteria encompassed other causes of elevated intracranial pressure, including primary brain tumors (ICD-10: C71.*), secondary brain metastases (ICD-10: C79.31), cerebral arteriovenous malformations (ICD-10: Q28.2), and venous sinus thrombosis (ICD-10: I67.6).

The study population was divided into two groups. The metformin group consisted of patients with IIH who received metformin (minimum dose 500mg daily) with no prior history of diabetes or prediabetes, and their first prescription of metformin was initiated after IIH diagnosis. The control group comprised patients with IIH who did not receive metformin at any point during the study period and had no prior history of diabetes or prediabetes. These groups were matched for age, sex, race, ethnicity, baseline body mass index (BMI), and baseline HbA1c using propensity score matching to minimize selection bias.

Primary outcomes were defined as papilledema (ICD-10: H47.1), refractory IIH status (ICD-10: G93.2 with modifier code Z91.82), and therapeutic spinal puncture rate (CPT: 62272). Secondary outcomes included optic atrophy (ICD-10: H47.2), blindness (ICD-10: H54.*), pulsatile tinnitus (ICD-10: H93.A9), diplopia (ICD-10: H53.2), visual field defects (ICD-10: H53.4), and adverse events related to metformin use.

We analyzed the data at different follow-up durations (one-month, three-months, six-months, 12-months, and 24-months) and assessed the following outcomes: papilledema, optic atrophy, blindness, pulsatile tinnitus, diplopia, refractory IIH status, visual discomfort, visual field defects, and therapeutic spinal puncture rate as the primary treatment. For outcome assessment purposes, refractory IIH was defined as persistent or worsening symptoms despite maximum medical therapy for three months or longer. Treatment success was characterized by the resolution of papilledema and improvement in visual function, while disease recurrence was defined as a new onset of symptoms after documented resolution. Therapeutic spinal punctures were distinguished from diagnostic procedures, specifically identifying lumbar punctures performed for therapeutic purposes. Visual outcomes encompassed any documented changes in visual acuity or visual fields that were measured as the change from baseline at specified time points.

## 3. Results

### 3.1. Baseline Demographics:

A comprehensive overview of the baseline demographics and clinical characteristics for patients with IIH is presented in Table 1, comparing metformin and control groups before and after propensity score matching. Initially, the cohorts comprised 1,268 patients in the metformin group and 49,262 in the control group, with notable disparities in several parameters. Post-matching, both cohorts were refined to 1,267 patients each, achieving remarkable comparability across baseline attributes. The mean age was nearly identical (36.8 *vs*. 37.0 years), with comparable standard deviations. Gender distribution revealed a striking female predominance (93.29% *vs.* 92.66%), consistent with the known epidemiology of IIH. Comorbidity profiles highlighted the complex medical landscape of IIH patients. Endocrine and metabolic diseases were highly prevalent (73.48% *vs.* 73.01%), potentially reflecting the metabolic dysfunction often associated with IIH. Notably, ophthalmological diseases affected approximately 59% of patients in both groups, underscoring the significant ocular manifestations in IIH. Other frequent comorbidities included musculoskeletal diseases, mental and neurodevelopmental disorders, and respiratory conditions, all showing similar distributions between groups.

### 3.2. Outcomes Analysis:

We performed a longitudinal outcome analysis between the metformin group and the control group in patients with IIH, and the results are presented in Table 2. The metformin group consistently demonstrated lower risk percentages for most outcomes compared to the control group. Papilledema and refractory IIH showed very high statistical significance (p<0.0001) in favor of the metformin group at all follow-up points (1, 3, 6, 12, and 24 months). The risk ratios for these outcomes ranged from 0.238 to 0.889, indicating a substantially lower risk in the metformin group. Optic atrophy risk was similar between the groups at 1, 3, 6, and 12 months, but at 24 months, the metformin group had a slightly higher risk (2.1% vs. 0.8%, p=0.047). Blindness risk was significantly lower in the metformin group at 3 months (p=0.031), but not statistically significant at other follow-up points. Pulsatile tinnitus and diplopia showed significantly lower risks in the metformin group at 6 months (p=0.005 and p=0.007, respectively) and 24 months (p=0.002 and p<0.0001, respectively). However, the differences were not statistically significant at one-month, three-months, and 12-months. Visual discomfort and visual field defects were significantly lower in the metformin group only at 3 months (p=0.025), with no significant differences at other follow-up durations. The therapeutic spinal puncture rate was significantly lower in the metformin group at all follow-up points (one-month, three-months, six-months, 12-months, and 24-months), with p-values ranging from 0.0001 to 0.007. The risk difference and risk ratio favored the metformin group across all durations, with significant p-values (p<0.0001). The 95% confidence intervals for the risk ratios indicated a consistent benefit of metformin over the entire study period.

### 3.3. Metformin Safety Profile:

We analyzed a total of 2,534 patients, equally divided between the metformin and control groups (1,267 patients each) after performing propensity score matching analysis for safety and side effects of metformin. Gastrointestinal side effects, often associated with metformin use, showed similar incidence rates in both groups. Notably, nausea was reported in 8.52% of metformin users compared to 10.58% in the control group (RR 0.81, 95% CI 0.63–1.03, p=0.09). Vomiting occurred less frequently, affecting 2.37% and 3.31% of the metformin and control groups, respectively (RR 0.71, 95% CI 0.45–1.13, p=0.19). Regarding metabolic side effects, lactic acidosis—a rare but serious concern with metformin use—was observed in 1.03% of metformin users versus 1.74% in the control group (RR 0.59, 95% CI 0.30–1.17, p=0.17). Vitamin B12 deficiency or megaloblastic anemia showed identical rates in both groups (4.58%, RR 1.0, 95% CI 0.70–1.43, p=0.999). General and systemic side effects were also comparable between groups. Myalgia was reported in 6.47% of metformin users and 8.29% of controls (RR 0.78, 95% CI 0.59–1.03, p=0.09), while asthenia affected 5.21% and 5.84% of the metformin and control groups, respectively (RR 0.89, 95% CI 0.65–1.23, p=0.54).

## 4. Discussion

In our large-scale multicenter retrospective study based on the TriNetX database, we illustrated compelling evidence for the potential efficacy of metformin as a disease-modifying therapy in IIH. Our findings demonstrate significant improvements across multiple IIH-related outcomes in patients treated with metformin compared to those who did not receive the medication.

The marked reduction in papilledema risk observed in the metformin group throughout the study period is particularly striking. This finding aligns with recent research suggesting that metformin may have direct effects on ICP regulation. Botfield et al. [13] demonstrated that metformin can reduce ICP in rodent models of IIH through AMPK-dependent inhibition of the Na+/K+-ATPase at the choroid plexus, thereby decreasing CSF secretion. Our clinical findings support this preclinical evidence, indicating that metformin’s effects on papilledema may be mediated through direct modulation of CSF dynamics rather than solely through weight loss.

The observed reduction in refractory IIH status among metformin-treated patients is particularly noteworthy. This finding suggests that metformin may address underlying pathophysiological mechanisms that contribute to treatment resistance in IIH. Recent evidence has implicated adipose tissue dysfunction and altered adipokine profiles in IIH pathogenesis [11]. Metformin’s known effects on adipose tissue function, including modulation of adipokine secretion and improvement of insulin sensitivity, may contribute to its efficacy in refractory cases. Furthermore, emerging evidence suggests that metformin can influence the gut microbiome, which has been increasingly linked to neurological disorders, including those affecting ICP regulation [19].

These multifaceted effects of metformin may explain its potential to improve outcomes in patients who have not responded adequately to conventional therapies. The latest evidence has highlighted the importance of metabolic dysfunction in IIH pathogenesis, independent of obesity. For instance, Hornby et al. demonstrated alterations in glucose and lipid metabolism in IIH patients that were not fully explained by BMI [20]. Metformin’s pleiotropic effects on metabolism, including improved insulin sensitivity and modulation of lipid profiles, may therefore contribute to its efficacy in IIH through mechanisms distinct from weight loss.

The potential endocrinological connections underlying metformin’s efficacy in IIH are particularly interesting. Recent studies have implicated various endocrine factors in IIH pathophysiology, including androgens, glucocorticoids, and growth hormones [21]. Metformin has been shown to influence several of these endocrine pathways. For example, metformin can reduce androgen levels and improve insulin sensitivity in polycystic ovary syndrome (PCOS), a condition often comorbid with IIH [22]. Given that androgen excess has been implicated in IIH pathogenesis, metformin’s androgen-lowering effects may contribute to its therapeutic benefits. Additionally, metformin has been shown to modulate the hypothalamic-pituitary-adrenal (HPA) axis, which could influence CSF dynamics and ICP regulation [23]. These endocrinological effects of metformin may explain, in part, its apparent disease-modifying properties in IIH observed in our study.

The safety profile of metformin in our IIH cohort was favorable, with no significant differences in adverse events compared to the control group. This is consistent with metformin’s well-established safety record in other clinical contexts and supports its potential as a long-term therapy for IIH. The similar incidence of lactic acidosis between the metformin and control groups is particularly reassuring, given historical concerns about this rare but serious complication [24].

Our findings have important clinical implications. The observed reductions in papilledema and refractory disease status suggest that metformin could address multiple aspects of IIH pathophysiology.

While our results are highly promising, several important limitations of this study warrant careful consideration. First, the retrospective nature of our analysis inherently introduces potential for selection bias and confounding factors, despite our rigorous propensity score matching approach. The use of electronic health record data, while providing a good sample size, may be subject to coding errors, missing data, or inconsistent documentation practices across different healthcare institutions within the TriNetX network. A significant limitation is the inability to directly measure intracranial pressure or access detailed CSF dynamics data. The absence of direct ICP measurements and CSF opening/closing pressures limits our ability to quantify the precise physiological effects of metformin on CSF dynamics. Additionally, we could not standardize the methods and timing of ophthalmological assessments across institutions, potentially introducing variability in the evaluation of visual outcomes.

The study’s reliance on ICD-10 codes for diagnosis and outcome measurement may not capture the full spectrum of disease severity or subtle clinical changes. Furthermore, while we controlled for various confounding factors, we cannot completely account for all potential confounders, such as dietary habits, exercise patterns, or concurrent medications that might influence IIH outcomes. The impact of these unmeasured variables on our results remains unknown.

Patient compliance with metformin therapy could not be definitively assessed beyond prescription fills, and we lacked data on medication adherence patterns. The study also cannot account for potential variations in clinical practice patterns across different institutions, including differences in the threshold for therapeutic interventions or the timing of treatment escalation. Another limitation is the potential for immortal time bias, as patients in the metformin group had to survive long enough to receive the prescription. While our matching process attempted to minimize this bias, its influence cannot be completely eliminated. Additionally, the study’s follow-up period, though substantial, may not be sufficient to capture very long-term outcomes or rare adverse events.

The generalizability of our findings may be limited by the study population’s characteristics and the participating healthcare institutions’ geographic and demographic distribution. Furthermore, the exclusion of patients with diabetes and pre-diabetes, while necessary for studying metformin’s direct effects on IIH, means our results may not be applicable to IIH patients with these comorbidities. Finally, as with any observational study, we can demonstrate association but not causation. The precise mechanisms by which metformin influences IIH outcomes remain speculative and require validation through prospective, mechanistic studies. These limitations underscore the need for randomized controlled trials to definitively establish metformin’s role in IIH management and elucidate its therapeutic mechanisms.

## 5. Conclusions

Our study provides strong evidence for the potential of metformin as a disease-modifying therapy in IIH, with benefits extending beyond weight loss. These findings open new avenues for IIH management and underscore the need for further research into the complex pathophysiology of this condition. Prospective, randomized controlled trials are now warranted to confirm these results and establish optimal treatment protocols. Such studies should include direct measurements of ICP, CSF opening pressure estimations, detailed ophthalmological assessments, and investigations in a longitudinal manner into the underlying mechanisms of metformin’s effects in IIH. Additionally, long-term follow-up studies will be crucial to assess the durability of metformin’s benefits and its impact on disease progression. As our understanding of IIH pathophysiology continues to evolve, metformin may represent a promising addition to the therapeutic armamentarium for this challenging condition.

## Figures and Tables

**Table 1:** Baseline Demographics of The Patients Cohorts.

Total Patients, n	Before Propensity Score Matching		P-Value	After Propensity Score Matching		P-Value
	Metformin Group	Control Group		Metformin Group	Control Group	
	1,268	49,262		1,267	1,267	
*Mean Age, SD*	36.8 ± 9.66	36.2 ± 10.1	0.0323	36.8 ± 9.66	37 ± 10	0.6114
**Sex, n (%)**						
*Female*	1,182, (93.22%)	41,006, (83.24%)	< 0.0001	1,182, (93.29%)	1,174, (92.66%)	0.5340
*Male*	53, (4.18%)	5,503, (11.17%)	< 0.0001	53, (4.18%)	54, (4.26%)	0.9213
*Unknown*	32, (2.52%)	1,705, (3.46%)	0.0537	32, (2.53%)	39, (3.08%)	0.3994
**Ethnicity, n (%)**						
*Not Hispanic or Latino*	847, (66.80%)	28,606, (58.07%)	< 0.0001	847, (66.85%)	859, (67.80%)	0.6113
*Hispanic or Latino*	129, (10.17%)	4,111, (8.35%)	0.0378	129, (10.18%)	126, (9.94%)	0.8430
*Unknown Ethnicity*	291, (22.95%)	15,479, (31.42%)	< 0.0001	291, (22.97%)	282, (22.26%)	0.6691
**Race, n (%)**						
*White*	730, (57.57%)	26,556, (53.91%)	0.0731	730, (57.62%)	726, (57.30%)	0.8723
*Black or African American*	232, (18.30%)	7,905, (16.05%)	0.0694	232, (18.31%)	246, (19.42%)	0.4771
*Other Race*	54, (4.26%)	2,271, (4.61%)	0.4567	54, (4.26%)	47, (3.71%)	0.4772
*Asian*	18, (1.42%)	712, (1.45%)	0.8702	18, (1.42%)	23, (1.82%)	0.4311
*American Indian or Alaska Native*	10, (0.79%)	161, (0.33%)	0.0064	10, (0.79%)	10, (0.79%)	0.9999
*Native Hawaiian or Other Pacific Islander*	10, (0.79%)	117, (0.24%)	0.0001	10, (0.79%)	0, (0.00%)	0.0015
*Unknown Race*	226, (17.82%)	10,492, (21.30%)	< 0.0001	226, (17.84%)	223, (17.60%)	0.6691
**Comorbid Diseases, n (%)**						
*Endocrine and Metabolic Diseases*	931, (73.42%)	15,748, (31.97%)	< 0.0001	931, (73.48%)	925, (73.01%)	0.7877
*Ophthalmological Diseases*	750, (59.15%)	20,859, (42.34%)	< 0.0001	750, (59.19%)	754, (59.51%)	0.8715
*Musculoskeletal Diseases*	677, (53.39%)	13,730, (27.87%)	< 0.0001	677, (53.43%)	658, (51.93%)	0.4497
*Mental and Neurodevelopmental Disorders*	664, (52.37%)	13,249, (26.89%)	< 0.0001	664, (52.41%)	650, (51.30%)	0.5778
*Respiratory Diseases*	581, (45.82%)	12,811, (26.01%)	< 0.0001	581, (45.86%)	581, (45.86%)	0.9999
*Genitourinary Diseases*	606, (47.79%)	10,356, (21.02%)	< 0.0001	606, (47.83%)	614, (48.46%)	0.7504
*Digestive Tract Diseases*	519, (40.93%)	10,079, (20.46%)	< 0.0001	519, (40.96%)	514, (40.57%)	0.8398
*Presence of Active Infections*	386, (30.44%)	6,770, (13.74%)	< 0.0001	386, (30.47%)	375, (29.60%)	0.6336
*Skin and Subcutaneous Diseases*	480, (37.85%)	6,729, (13.66%)	< 0.0001	480, (37.88%)	483, (38.12%)	0.9023
*Circulatory Diseases*	261, (20.58%)	5,944, (12.07%)	< 0.0001	261, (20.60%)	270, (21.31%)	0.6604
*Hematological and Immunological Diseases*	279, (22.00%)	5,683, (11.54%)	< 0.0001	279, (22.02%)	252, (19.89%)	0.1875
*Active Malignancies (Excluding CNS Tumors and Brain Metastasis)*	257, (20.27%)	4,072, (8.27%)	< 0.0001	257, (20.28%)	238, (18.78%)	0.3411
*Presence of Congenital Malformations or Chromosomal Abnormalities*	112, (8.83%)	1,955, (3.97%)	< 0.0001	112, (8.84%)	105, (8.29%)	0.6192

Abbreviations: CNS: Central Nervous System

**Table 2:** Comparison Between Outcomes and Their Follow-up Duration Between Both Groups Through Propensity Score Matching.

Outcome	Follow-up Duration	Metformin Group Risk Percentage	Control Group Risk Percentage	Risk Difference	Risk Ratio	95% Confidence Interval	P-value
**Papilledema**	1-month	2.80%	11.60%	−8.80%	0.238	(0.166, 0.341)	**0.0001** ***
	3-months	6.70%	16.70%	−10.00%	0.401	(0.316, 0.509)	**0.0001** ***
	6-months	9.50%	19.40%	−10.00%	0.488	(0.398, 0.598)	**0.0001** ***
	12-months	11.00%	19.90%	−8.90%	0.553	(0.457, 0.668)	**0.0001** ***
	24-months	12.40%	21.60%	−9.20%	0.573	(0.479, 0.686)	**0.0001** ***
**Optic Atrophy**	1-month	0.80%	0.80%	0.00%	1.0	(0.418, 2.394)	0.999
	3-months	0.80%	0.80%	0.00%	1.0	(0.418, 2.394)	0.999
	6-months	0.90%	0.80%	0.20%	1.2	(0.520, 2.767)	0.668
	12-months	1.50%	1.40%	0.10%	1.056	(0.557, 2.002)	0.868
	24-months	1.70%	0.80%	0.90%	2.1	(0.993, 4.441)	**0.047** *
**Blindness**	1-month	0.80%	1.30%	−0.50%	0.625	(0.285, 1.372)	0.237
	3-months	0.90%	2.00%	−1.00%	0.48	(0.242, 0.951)	**0.031** *
	6-months	1.50%	2.10%	−0.60%	0.704	(0.393, 1.259)	0.234
	12-months	1.80%	2.80%	−1.00%	0.639	(0.381, 1.072)	0.087
	24-months	2.10%	2.60%	−0.60%	0.788	(0.474, 1.309)	0.356
**Pulsatile Tinnitus**	1-month	0.80%	0.90%	−0.20%	0.833	(0.361, 1.922)	0.668
	3-months	0.80%	1.50%	−0.70%	0.526	(0.246, 1.127)	0.093
	6-months	0.80%	2.10%	−1.30%	0.37	(0.180, 0.762)	**0.005** **
	12-months	1.10%	1.70%	−0.60%	0.636	(0.327, 1.238)	0.179
	24-months	1.10%	2.80%	−1.70%	0.40	(0.216, 0.740)	**0.002** **
**Diplopia**	1-month	0.80%	1.30%	−0.50%	0.625	(0.285, 1.372)	0.237
	3-months	0.80%	1.80%	−1.00%	0.435	(0.208, 0.910)	0.023
	6-months	0.80%	2.10%	−1.30%	0.385	((0.186, 0.794)	**0.007** **
	12-months	0.80%	1.80%	−1.00%	0.435	(0.208, 0.910)	**0.023** *
	24-months	0.80%	2.80%	−2.10%	0.278	(0.138, 0.557)	**0.0001** ***
**Refractory IIH**	1-month	16.70%	30.60%	−14.00%	0.544	(0.469, 0.631)	**0.0001** ***
	3-months	31.40%	42.70%	−11.40%	0.734	(0.662, 0.814)	**0.0001** ***
	6-months	39.70%	49.50%	−9.90%	0.801	(0.733, 0.874)	**0.0001** ***
	12-months	45.90%	53.90%	−8.00%	0.851	(0.787, 0.920)	**0.0001** ***
	24-months	50.10%	56.30%	−6.20%	0.889	(0.826, 0.957)	**0.002** **
**Visual Discomfort and Visual Field Defects**	1-month	0.90%	1.70%	−0.80%	0.524	(0.254, 1.082)	0.075
	3-months	1.40%	2.70%	−1.30%	0.529	(0.301, 0.932)	**0.025** *
	6-months	2.20%	3.50%	−1.30%	0.636	(0.399, 1.016)	0.056
	12-months	3.30%	3.70%	−0.40%	0.896	(0.598, 1.342)	0.594
	24-months	3.80%	4.40%	−0.60%	0.857	(0.588, 1.250)	0.423
**Therapeutic Spinal Puncture Rate**	1-month	0.80%	2.20%	−1.40%	0.357	(0.174, 0.732)	**0.003** **
	3-months	0.80%	2.40%	−1.70%	0.323	(0.159, 0.655)	**0.001** **
	6-months	0.80%	2.70%	−1.90%	0.294	(0.146, 0.593)	**0.0001** ***
	12-months	0.90%	2.20%	−1.30%	0.414	(0.212, 0.807)	**0.007** **
	24-months	1.20%	3.10%	−1.90%	0.385	(0.213, 0.694)	**0.001** **

* Denotes Statistical Significance

** Denotes High Statistical Significance

*** Denotes Very High Statistical Significance

## Data Availability

Available on TriNetX Database Based on Institutional Collaborations.
